# Tensile Behavior of Carbon Fibers Impregnated with Thermoplastics Using Coextrusion Technique

**DOI:** 10.3390/polym18050651

**Published:** 2026-03-06

**Authors:** Victor V. Tcherdyntsev, Andrey A. Stepashkin, Alnis A. Veveris

**Affiliations:** Laboratory of Functional Polymer Materials, National University of Science and Technology “MISIS”, Leninskii Prosp, 4, 119049 Moscow, Russia; a.stepaskin@misis.ru (A.A.S.); mrechoru48@gmail.com (A.A.V.)

**Keywords:** carbon fibers, coextrusion, impregnation, polypropylene, ethylene vinyl acetate, tensile behavior

## Abstract

To increase printing speed and quality, a route consisting of using two sequential coextruders to form impregnated fiber immediately before feeding it to the printer. Such an approach, aimed at allowing the use of the most common industrial 12K carbon fibers for additive manufacturing, prevents damage to composite fibers during transportation, storage, and loading. An elaborate system was used to prepare carbon fibers impregnated with polypropylene, ethylene vinyl acetate, and their blends. The used scheme allows the production of composite fibers containing from 60 to 80 wt. % of carbon fibers. It was found that the elastic modulus of the composite fibers is close to those for raw carbon fibers and does not depend on the used polymer. It shows that the used carbon fiber path in the polymer melt and two sequential calibrating nozzles result in a high degree of orientation of the elementary filaments in the fiber at impregnation and maintain the elastic properties of the carbon fiber in the resulting composite. The tensile strength of the composite fibers depends on the polymer content in the composite fiber; the highest tensile strength was observed for fibers impregnated with ethylene vinyl acetate when increasing the coextrusion temperature up to 220 °C, which results in a composite fiber with a polymer content of 30 wt. %. A decrease in the polymer content in composite fibers results in a decrease in strength.

## 1. Introduction

Additive manufacturing encompasses a large group of rapidly evolving methods for the direct creation of finished products that are fundamentally different from traditional methods such as casting or extrusion. Since its inception in the early 1980s, additive manufacturing has evolved significantly, moving from a conceptual stage to the creation of widely used unique products or small-batch production series. In the additive manufacturing of polymer materials, the most widely used additive manufacturing technology is fused filament fabrication (FFF), which is based on the hot extrusion of a polymer filament. The widespread use of FFF is associated with the development of the RepRap project in 2005, which has become the basis for most modern 3D printers [1,2,3,4].

However, there are several restrictions limiting the application of the FFF technique. One of them is associated with the low strength and performance characteristics of the used polymers, such as acrylonitrile butadiene styrene (ABS), polylactide (PLA), polyethylene terephthalate glycol (PET-G), polypropylene (PP) and ethylene vinyl acetate (EVA). These polymers are widely used in the FFF process due to their low processing temperatures and low cost, but their low strength does not allow the creation of functional products operated under load [5,6,7,8,9].

The design of fully functional products for industrial applications currently relies on the use of engineering thermoplastics, such as polycarbonate (PC) and various polyamides (PAs). The latter dominates industrial applications but is characterized by low chemical resistance and high water absorption, which significantly reduces the level of properties when in contact with atmospheric moisture. The use of PC is limited by its high coefficient of thermal expansion and the resulting thermal deformation and differentiation of finished products. Of greatest practical interest is the production of products made from superstructural thermoplastics, such as polyarylene ether ketone (PAEK), polyphenylene sulfide (PPS), and polyesterimide (PEI), via 3D printing. These materials possess excellent strength, rigidity, chemical and abrasive resistance, and thermal properties. The use of these polymers presents a number of challenges because most commercially available printers are not designed to work with them, and the polymers themselves often have significant adhesion to metals and glass, which are often found in printer designs [10,11,12,13].

Utilizing common thermoplastic reinforced with dispersed fillers to produce filaments suitable for 3D printing is considered an alternative to the application of expensive engineering and superstructural polymers. Dispersed particles can be easily introduced into the polymer matrix during filament production. Such an approach allows the improvement of the mechanical characteristics of common polymers; moreover, filled material shows less shrinkage and reduced warping during printing. The static and dynamic coefficients of elasticity, wear resistance, permittivity, and thermal conductivity of such composites can be significantly increased in relation to unfilled polymers. Increased thermal conductivity reduces internal thermal stress and associated deformation in the product, a factor that significantly limits the use of unfilled polymers in engineering and superstructure applications. However, using dispersed fillers results only in a slight increase in mechanical properties, typically about 10%, which limits the use of dispersed filled polymers in structural applications [14,15,16,17].

To significantly improve the mechanical properties of polymer composites, fiber reinforcers, such as glass or carbon fibers, can be used. Short (up to 3 mm in length) fiber reinforcement allows the formation of a composite using traditional methods, such as injection molding, and is actively being adopted in additive manufacturing. Using polymers reinforced with short fibers in additive manufacturing allows significant improvement of the mechanical properties of the material. Using short fibers as reinforcers results in durable, stiff, impact- and wear-resistant materials that do not require expensive tooling or technical operations. The key factors determining the final properties of such composites are the fiber length and the interface adhesion. When the composite is destroyed, short fibers are not destroyed but instead pulled out of the matrix; this is associated with low adhesion of thermoplastics to the fiber surface and is addressed through fiber processing and polymer functionalization. Additionally, such composite properties are affected by the uniformity of fiber distribution over the matrix volume and length of the composite filaments [17,18,19,20,21,22,23].

Even more significant improvements in the mechanical properties of polymer composites can be achieved using filaments with continuous reinforcing fibers in printing. This allows for the creation of complex product geometries with minimal time and waste. Using continuous fibers and tapes, the material properties of the product can be optimized according to its operating conditions by controlling the anisotropy of the material within a single layer and throughout the entire structure, ensuring the desired combination of strength, stiffness, and other performance characteristics. Automated placement systems for continuous fibers and unidirectional tapes are a common industrial solution, implemented using robotic arms. However, the size, complexity, and cost of these systems prevent small-scale production, thus necessitating alternative technologies for producing small-sized polymer parts reinforced with continuous fibers. Additive manufacturing, primarily the FFF process, currently offers an alternative [10,24,25,26,27,28,29].

Carbon-based materials, including continuous carbon fibers, are widely used as reinforcers in polymer composites design [30,31,32]. Application of continuous carbon fibers in the FFF process significantly improves the elastic and strength properties of the resulting materials, significantly exceeding those of discrete-filled materials. The development of continuous fiber filament printing technology has been driven by advancements in related equipment, such as FFF printers from Markforged, Anisoprint, and others. Markforged FFF printers can manufacture parts using nylon filled with discrete or continuous carbon fiber. The content of continuous glass or carbon fibers in such composite filaments is approximately 30–40% by volume. Parts produced using these printers with continuous carbon fibers in the filament exhibit tensile strengths of ~1000 MPa and elastic moduli of 64 GPa, significantly exceeding the performance of materials with short fibers [33,34,35,36,37,38,39]. Anisoprint LLC’s widely available printers have also achieved similar performance levels for materials produced using the FFF process, using PA, PETG, PC, PEEK, and PEI matrices [40,41,42].

Even a small amount of continuous carbon fibers in composites can significantly improve their mechanical properties. As it was reported in [43], at a carbon fiber content in a PLA matrix of ~6 wt. %, the tensile modulus and strength of the 3D-printed sample reached ~19 GPa and ~185 MPa, respectively, which is a 599% and 435% increase in the same values for pure PLA. Impregnation of carbon fibers containing 1000 elemental filaments with PLA resulted in a maximum fiber content of 27% by volume in the composite, whereas the flexural strength and elastic modulus of the composite were 335 MPa and 30 GPa, respectively [43]. The printhead temperature also significantly affected the strength properties. Changing the temperature from 180 °C to 240 °C increased the elastic modulus from 5.4 to 8.6 GPa and the flexural strength from 115 to 155 MPa, respectively; this is associated with the increase in melt flow rate, which improves the quality of the impregnation process.

The weak adhesion between carbon fibers and thermoplastic polymers significantly affects the properties of the resulting composites. Surface modification of carbon fiber can enhance the bond strength between the fibers and the polymer, thereby improving the properties of the resulting products. It was observed [44] that the tensile and flexural strengths of the modified carbon fiber reinforced composites are 13.8% and 164% higher, respectively, than those of the original carbon fiber reinforced composites. Therefore, surface modification of the fibers is an effective tool for controlling properties. Typically, the fiber filling level of printed samples does not exceed 10–25 vol. %, but some papers [45] report the feasibility of producing highly filled (42.32 and 51.92 vol. % fiber) PLA-based carbon fiber-reinforced plastics using FFF printing.

Currently, 3D printing filaments containing continuous carbon fibers are produced using filaments containing 1000, 1500, and, very rarely, 3000 elementary filaments. The market share of these carbon fibers is extremely small. A second major challenge is the limited availability of polymers used in finished, mass-produced products, such as filaments containing continuous carbon fibers. A third important issue is that pre-produced filaments can be damaged during storage and transportation due to the high rigidity of carbon fibers, which reduces the properties of the materials produced using them. Therefore, our proposed innovation involves producing the printing filament immediately before the printing process. This allows us to produce filaments with the polymer required for a specific solution and utilizes the most common 12K fibers (or those containing 12,000 elementary filaments). These filaments allow for the formation of a larger volume of composite material per unit of time, increasing process efficiency.

Furthermore, the use of our proposed coextrusion system allows for the use of a wider range of thermoplastic polymers. Blends based on polypropylene and ethylene vinyl acetate are considered promising for the creation of composites, including those modified with nanoparticles [46,47]. It should be noted that the feasibility of printing with polypropylene and ethylene vinyl acetate has been demonstrated in [48].

Recently [49], we reported the development of a coextrusion system consisting of two sequential impregnation modules, each equipped with two heating systems, designed for integration into a 3D printer to form carbon yarns impregnated with thermoplastics in situ. It was observed that the system allows the formation of impregnated yarns with a high content of fibers and low defect density using standard 12K carbon fibers. The obtained impregnated yarn shows good tensile behavior, indicating that the rigidity of the raw carbon fiber was almost completely realized in the composite filaments. In [49], we used UMT49 12K-EP carbon fibers with a tensile strength of 4.9 GPa, an elastic modulus of 260 GPa, and an elongation at break of 1.8%. In the present investigation, Toray T700SC-12K carbon fiber, widely used for creating high-strength composite materials, was employed. These fibers have a tensile strength and elastic modulus close to those used in the previous study, but the elongation-at-break magnitude of T700SC-12K is noticeably higher than that of UMT49 12K-EP (2.1 vs. 1.8%). The carbon fibers used in the present study are of considerable interest due to their widespread use and commercial availability.

## 2. Materials and Methods

### 2.1. Materials

We selected polypropylene as the matrix material for the 3D printing filaments used in this study. Polypropylene is one of the most widely used thermoplastic polymers, offering high chemical resistance and inertness. The melt flowability of polypropylene can vary widely, so we used two grades with different melt flow rates. We selected PP H030 GP and PP H270 FF from PAO Sibur, Moscow, Russia, as the matrix for the resulting 3D printing filaments.

To control adhesion to various substrates, we examined Evatane EVA28-40, an ethylene vinyl acetate (EVA) copolymer (28% vinyl acetate content), manufactured by SK Functional Polymer, Seoul, Republic of Korea. This polymer exhibits significant adhesion to a wide range of supports due to its functional groups.

Toray T700SC-12K carbon fiber (Toray Industries, Inc., Tokyo, Japan), widely used for creating high-strength composite materials, was employed in this study. It has a high tensile strength of 4.9 GPa, an elastic modulus of 230 GPa, and an elongation at break of 2.1%.

### 2.2. Preparation of Composite Filaments

A pilot system for two-stage coaxial filament extrusion, described in [49], was used to obtain the impregnated filaments. The system includes two cylindrical brass impregnation tanks, heated by 500 W nozzle heaters mounted on them, similar to those used in injection molding machines. The melt temperature was monitored using Chromel–Alumel thermocouples. The upper bath was designed to impregnate the dried filament with polymer as it passed through the melt. The fiber path was 200 mm long, transitioning from a smooth 20 mm inner diameter to an M6 threaded hole on the lower surface, designed to accommodate E3d V6 nozzles as replaceable molds.

As the carbon filament is drawn through the upper bath, it is initially impregnated with the polymer melt, and excess polymer is removed through a standard 3D printing nozzle. However, droplets of excess polymer remain on the impregnated filament, so a second spinneret is used to improve impregnation quality, remove droplets, and achieve the final filament diameter. It also includes a 25 mm diameter, 50 mm long brass cylinder with a 20 mm tapered bore that tapers to a 2 mm wide channel, featuring an M6 threaded hole for die connection. The heater is similar to the one in the upper tank, but with a power of 200 W and a length of 30 mm. K-type thermocouples connected to the heater are used to measure the nozzle and melt temperatures.

**Figure 1:**
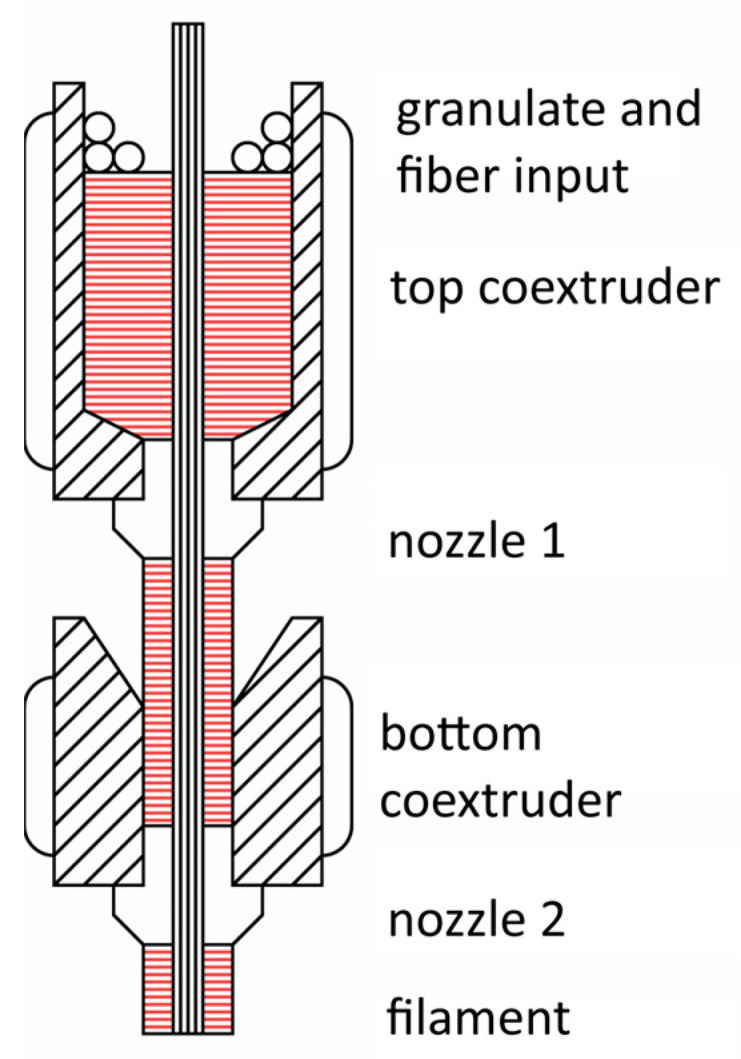
Scheme of impregnation setup.

A scheme of the impregnation setup is shown in Figure 1. To produce impregnated filament samples, 12k carbon filaments are passed through the die, polymer granules are loaded into the upper melt pool, and the heater is controlled to set the desired melt and coextrusion nozzle temperatures. After the polymer melts and the melt and components reach the extrusion system, the carbon filaments are drawn through the polymer melt. The drawing speed is set at 10 mm/s. It should be noted that we investigated the possible impact of drawing speed on the obtained results, which showed that differences in properties of fibers obtained at a speed of 10 mm/s and at lower speeds were statistically insignificant. For the design we used, a speed close to 10 mm/s is the maximum at which it is possible to produce high-quality filament for printing; at higher speeds, the quality of impregnation within the filament deteriorates. Generally, the drawing speed can be increased, but this will require an increase in the filament impregnation path to ensure the required quality.

Future plans include automating the process of feeding polymer granules into the extruder for continuous printing. The resulting filaments have a diameter of 1 mm, yielding filaments up to 12–15 m per cycle.

In the present study, we obtained six series of thermoplastic composite filament samples filled with continuous Toray T700SC-12K carbon fiber:

- PP H030 series, based on low-flow polypropylene grade H030, with a processing temperature of 220 °C;
- PP H270 series, based on high-flow polypropylene grade H270, with a processing temperature of 220 °C;
- EVA 28-40 (220) series, based on ethylene vinyl acetate copolymer grade 28–40, with a processing temperature of 220 °C;
- EVA 28-40 (190) series based on ethylene vinyl acetate copolymer grade 28–40, with a processing temperature of 190 °C;
- PP H270 50% EVA series based on a 1:1 blend of polypropylene H270 and ethylene vinyl acetate 28–40, with a processing temperature of 190 °C;
- PP H270 25% EVA series based on a 3:1 blend of polypropylene H270 and ethylene vinyl acetate 28–40, with a processing temperature of 220 °C;

### 2.3. Investigation

The microstructure of the specimens and fracture surfaces was examined using a TESCAN VEGA Compact (Joint stock company, Tescan, Brno, Czech Republic) and a Hitachi TM-1000 (Hitachi Ltd., Tokyo, Japan). Scanning electron microscopy in both Compo BSE and SE modes allowed us to gather information about the sample surface structure.

**Figure 2:**
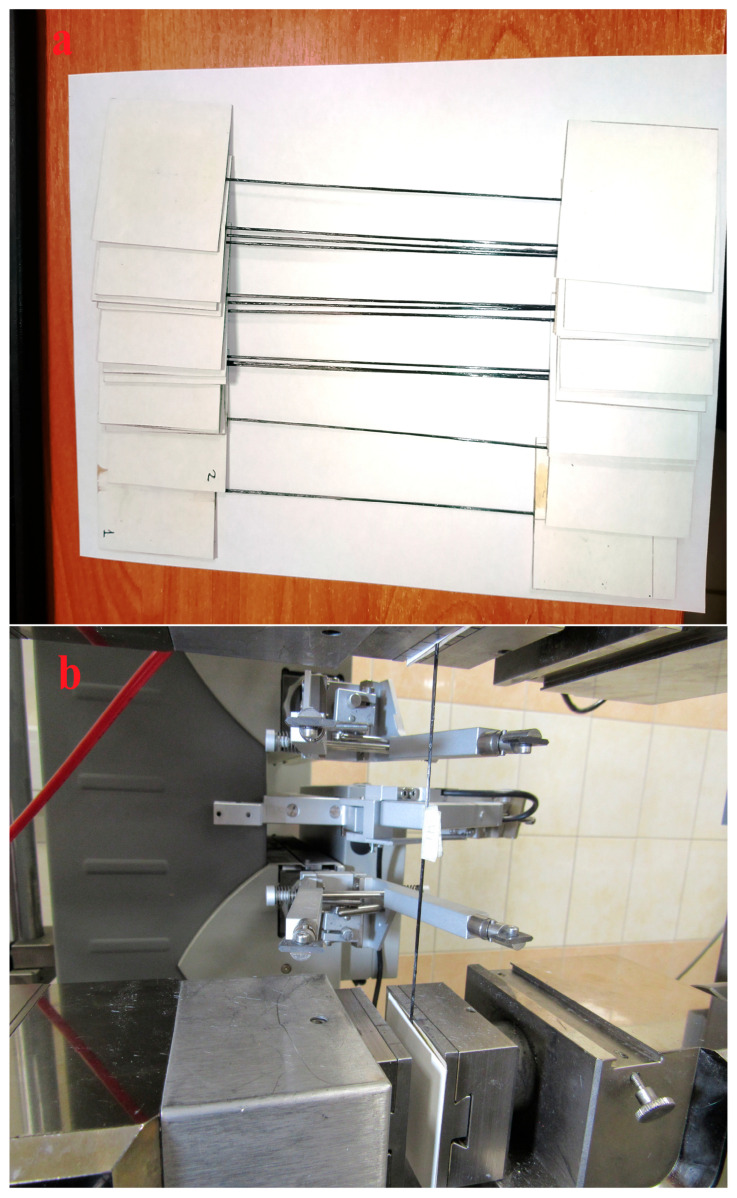
Samples for tensile test (**a**) and the process of tensile test of filament samples (**b**).

For tensile testing, 220 mm long specimens were cut from the obtained 3D-printed filaments, with a working length of 100 mm, and sealed at both ends to 1 mm thick protective cardboard, dimensions of cardboard was 52 × 60 mm. The specimens were secured in the cardboard cover using EHD epoxy adhesive (TU2225-607-11131395-2003, Epoxide LLC, Dzerzhinsk, Russia) and diethylenetriamine curing agent (CAS No. 111-40-0, Tosol Sintez Invest LLC, Dzerzhinsk, Russia). The epoxy resin was polymerized in an adhesive FD-115 drying oven (BINDER GmbH, Tuttlingen, Germany) at 80 °C for 2 h, followed by post-curing at 120 °C for 2 h. Samples prepared for testing are shown in Figure 2a.

Before sealing each sample in a cardboard frame, the polymer and carbon fiber content were determined using an AN and GR202 analytical balance (AND, Tokyo, Japan). The polymer content in the sample was determined based on the difference between sample mass and fiber mass, calculated based on linear density and sample length.

The strength and deformation properties of the obtained thermoplastic impregnated carbon fibers were determined using a Zwick/Roell z020 universal tensile testing machine (Zwick/Roell Group, Ulm, Germany) with a maximum applied force of 20 kN, equipped with a MultiXtens high-precision contact strain measurement system. Sample testing was performed according to ASTM D4018 [50] and ISO 10618 [51]. Sample setup and tensile testing illustrations are shown in Figure 2b.

To measure deformation during testing, a MultiXtens system (Zwick/Roell Group, Ulm, Germany) was used. It uses a contact probe with a polymer or steel tip to measure the longitudinal deformation of the specimen with an accuracy of 0.2 μm, while maintaining a constant clamping force on the specimen.

The specimen is clamped in a tensile testing machine using a pneumatic clamp, maintaining a constant closing force and cylinder pressure of 2 bar. The working length of the clamp thread is 100 mm, and the active clamping speed varies from 1 to 100 mm/min during the test. The distance between the sensor measurement points is 70 mm. To reduce the influence of clamp stress on the specimen, a protective cardboard pad protrudes 2–3 mm above the clamp surface. A total of 25 specimens were tested for a single result. Before any test, the specimens were conditioned for 88 h at a standard atmosphere of 23/50 according to ISO 291 [52].

The melting temperature and range during the heating and cooling crystallization process were determined using differential scanning calorimetry. This study was conducted using a NETZSCH DSC204 Phoenix f1 differential scanning calorimeter (Netzscg GmbH, Selb, Germany), conforming to ISO 11357-1 [53] s, and ISO 11357-3 [54]. The study was conducted under an inert gas atmosphere (argon) with a heating and cooling rate of 5 K/min. Polymer samples weighing 20–25 mg were sealed in aluminum crucibles. Measurements were performed in modes of heating to 240 °C, cooling to 30 °C, and reheating to 240 °C. Analysis of the DSC curves allowed us to investigate the range and peak melting and crystallization points of the thermoplastic polymers used in this study to select appropriate extrusion modes. The porosity of the yarn material was assessed using a Densi 100 Automatic True Density Analyzer (Altamira Instrument LLC., Cumming, GA, USA) using helium as the working gas.

## 3. Results and Discussion

The melting and crystallization temperature ranges of the polymers and polymer blends used for filament fabrication were investigated using DSC. We chose a test pattern consisting of initial heating, cooling, and second heating, as it is best suited to the thermal processes in the coextrusion system. During initial heating, two endothermic melt peaks were observed, likely due to the presence of crystals of varying sizes in the original polymer. During repeated heating, a single melt peak was observed, indicating that the polymer state stabilized upon cooling. PP H030 polypropylene also exhibited two melt peaks, and its behavior during the DSC study was similar to that of PP H270 polypropylene. The melting and crystallization temperatures of the studied polymers are listed in Table 1.

Polypropylene and ethylene vinyl acetate form completely immiscible polymer blends, as evidenced by completely separate melt and crystallization peaks observed in blends selected at 50/50 and 75/25 wt. % ratios. However, one polymer had no effect on the melting or crystallization of the other; no changes in the positions of the melt and crystallization peaks were observed in the DSC curves. The melt flow indices of the polymers are listed in Table 1. Based on the DSC results and recommendations for processing polypropylene, we selected 190 and 220 °C as the primary operating temperatures for filament production.

**Figure 3:**
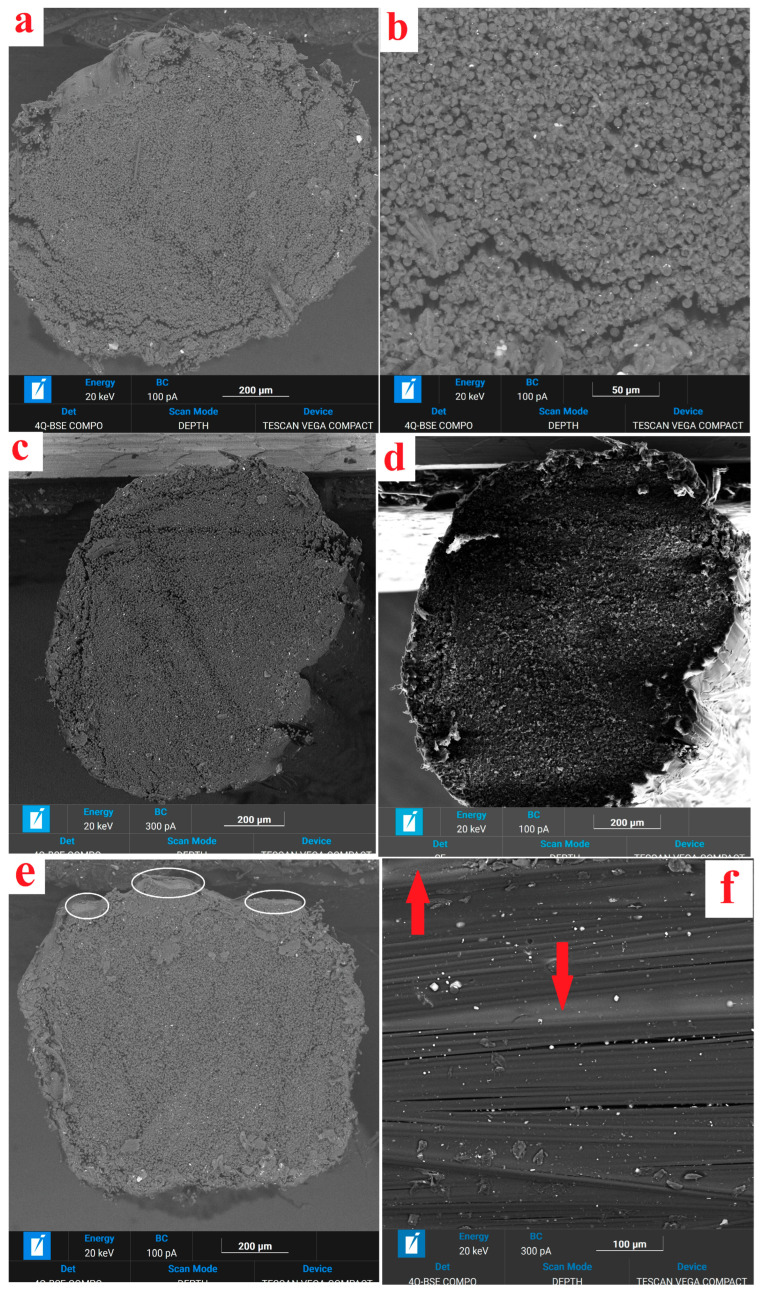
SEM image of structure of carbon fiber impregnated with PP H030 (**a**,**b**) (cross-sectional structure), PP H270 + 25% EVA (**c**–**e**) (cross-sectional structure), PP H270 + 25% EVA (**f**) (longitudinal sections). Polymer film is indicated with ellipses (**e**) and with arrows (**f**).

Figure 3 shows the SEM image of the cross-sectional and longitudinal structure of the obtained filaments. Long fiber path length in the polymer melt ensures uniform impregnation across the cross-section and the absence of polymer intercalations and large pores in the filaments, with the polymer content maintained at 25–30% by weight. When carbon filaments are impregnated with a PP + EVA blend, a polymer film of 10–15 μm in thickness is present on the fiber surface; see Figure 3e,f.

An analysis of SEM images shows that the resulting threads contain a small number of defects (Figure 3a,c) within them; they are virtually free of interlayers of pure polymer. At the same time, on the surface of the resulting threads (Figure 3e), we do not observe thick layers of polymer, which are removed during passage through the calibrating die, so it can be concluded that all the polymer is uniformly distributed within the thread. The amount of polymer is not optimal for obtaining a composite material during printing and amounts to 20–25% by weight, but the use of an additional thread of pure polymer allows us to eliminate this drawback. The main type of defect in the threads affecting the properties of the resulting materials is the “length difference” (misorientation) of individual bundles of elementary fibers in the thread, which is clearly visible in Figure 3f. Such misorientation in polysulfone/carbon fiber composites was studied in detail in [55].

**Figure 4:**
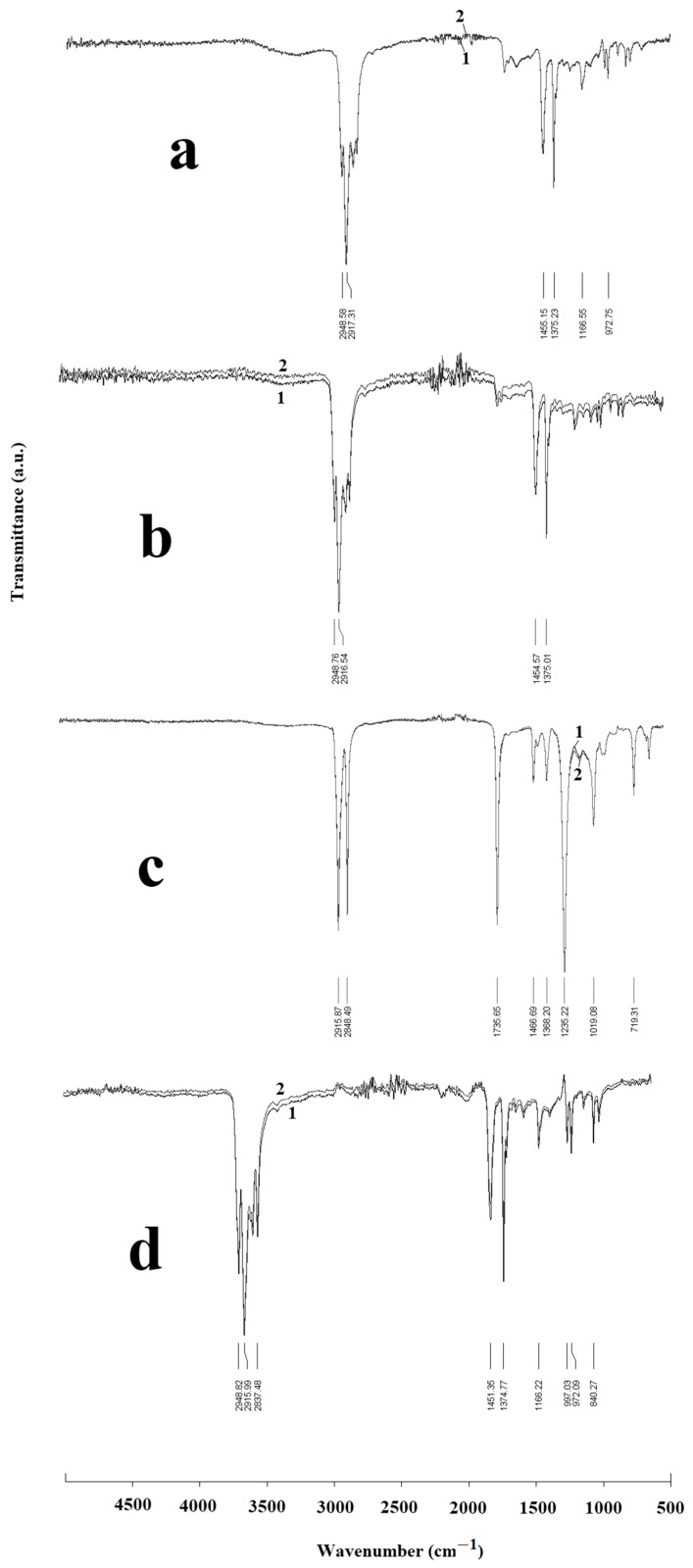
FTIR spectra of the initial (1) and oxidized (2) polymer samples. (**a**)—PP H030, (**b**)—PP H270, (**c**)—EVA, (**d**)—PP H270 + 25% EVA.

The oxidation of polypropylene and ethylene vinyl acetate during heating and extrusion was analyzed using FTIR. The samples for oxidation testing were collected after removing carbon filaments from the lower calibration matrix. A comparison of the spectra of the original (1) and oxidized samples (2) is shown in Figure 4. The polypropylene spectra show characteristic lines at approximately 2948 cm^−1^ and 2917 cm^−1^, which are associated with asymmetric stretching of C–H bonds. The peaks at 1455 cm^−1^ and 1375 cm^−1^ relate to asymmetric and symmetric vibrations in the CH_2_, while lines at 998 and 976 cm^−1^ depict vibrations of the C–C bonds (Figure 4a,b). In addition to slightly shifted lines similar to those in PP, the spectra of EVA contain lines associated with vibrations of the carbonyl groups –C=O at 1735 cm^−1^ and the deformation vibrations of the C–O bonds at 1235 cm^−1^ (Figure 4c). The PP/EVA blends show all the peaks of the raw polymers; no new lines were observed. This indicates the absence of interaction between EVA and PP, which confirms SEM and DSC observations and is consistent with the literature data [48,56,57]. The absence of changes in the lines at 1455 cm^−1^ and 1375 cm^−1^ in the spectra of PP/EVA blends compared to pure polymers was also observed in [58], where it was shown that the changes in the vibrations of the carbonyl group are observed only in the mixed blends.

No significant changes in the spectra caused by oxidation of the polymers were detected during their passage through the impregnation baths, as evidenced by a slight increase in the height of the peaks at 1735 cm^−1^ and 1235 cm^−1^. No new lines from other oxygen-containing functional groups formed during oxidative degradation located above 3000 cm^−1^ were observed. Lines at 3412 and 3347 cm^−1^, associated with hydrogen-bonded hydroperoxide and hydroxyl groups, were not observed in the FTIR spectra of polymers after extrusion.

Open porosity values are presented in Table 2. The open porosity P calculated from the measurement results for the materials is similar, ranging from 11 to 13%. No large pores are visible in the micrographs (Figure 3), suggesting that these are small pores oriented along the filament drawing directly.

**Figure 5:**
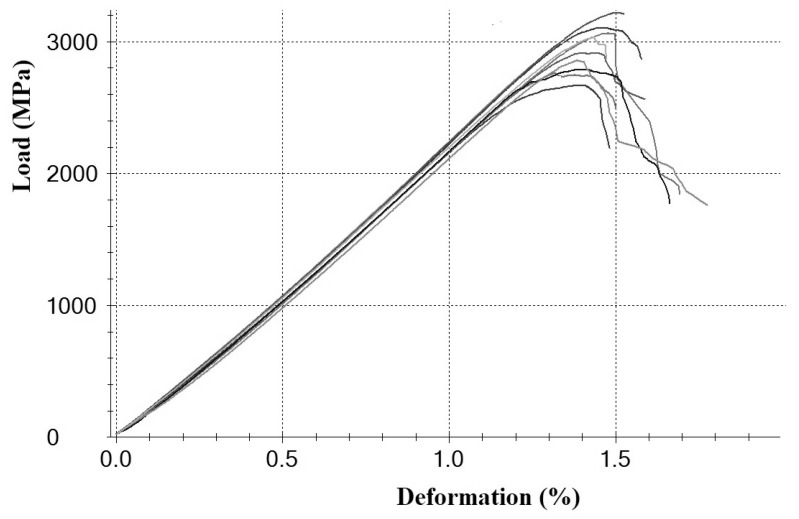
Load–deformation tensile curves for a series of composite filaments impregnated with EVA 28-40 (220 °C).

**Figure 6:**
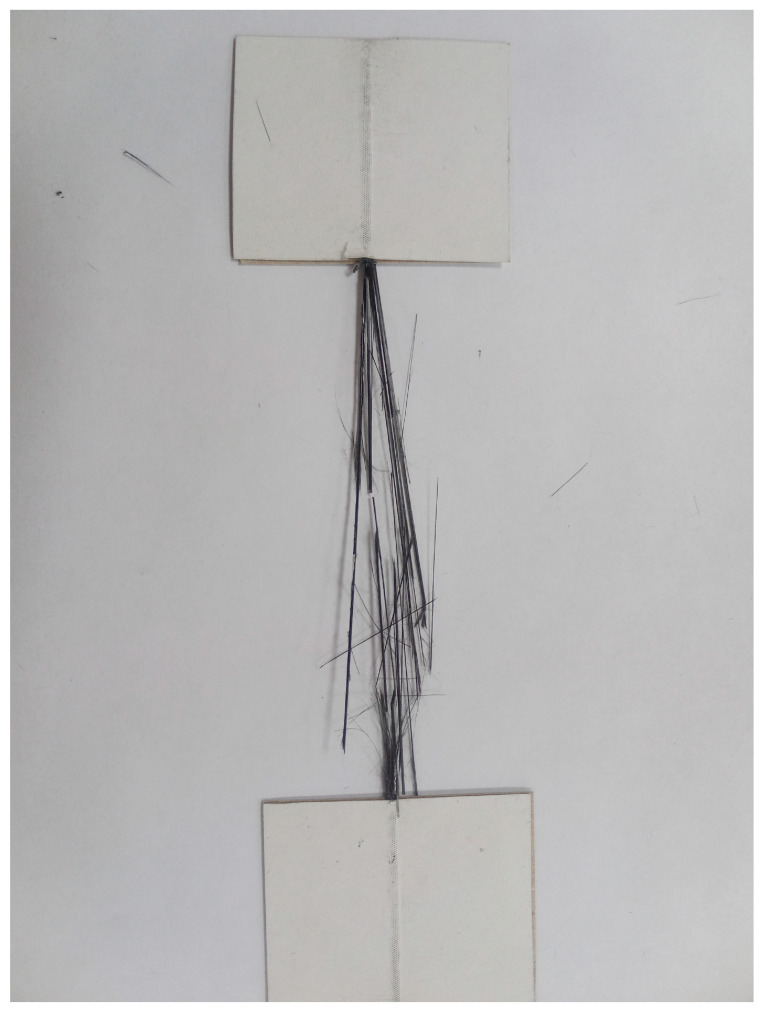
Appearance of filament composite destruction.

The filament strength within the test series shows a small dispersion, indicating a high degree of uniformity in the resulting strands. A typical load–strain relationship for a series of specimens is shown in Figure 5. This diagram is slightly nonlinear up to stresses approaching 90–95% of the failure stress. The tensile behavior of the filaments obtained in this study is given in Table 2, and the typical failure appearance is shown in Figure 6. It should be noted that the obtained magnitudes are close enough to those obtained [49] using UMT49 12K-EP carbon fibers and reveal the same tendency with varying polymer and impregnation temperature. It is evident that the behavior of different grades of carbon fibers is similar because they are high-strength fibers with similar standard properties, such as a tensile strength of 4.9 GPa and an elastic modulus of 230–240 GPa, and they contain the same number of elementary fibers in the filament structure, equal to 12,000. A second important factor is the deformation behavior of the identical thermoplastic matrix. Unlike epoxy binders, where elastic deformation occurs at stresses close to failure, due to the low deformation of epoxy resins, thermoplastic matrices begin to flow plastically at stresses of 300–500 MPa and strains of about 0.3–0.4%. This leads to the alignment of individual bundles of elementary fibers in the filaments and, consequently, an increase in the elastic modulus to values close to the rated values for a given fiber grade. Moreover, the observed deformation behavior does not depend on the type of fiber and the type of thermoplastic matrix [59,60], as well as the technology for producing impregnated threads through a solution or polymer melt.

**Figure 7:**
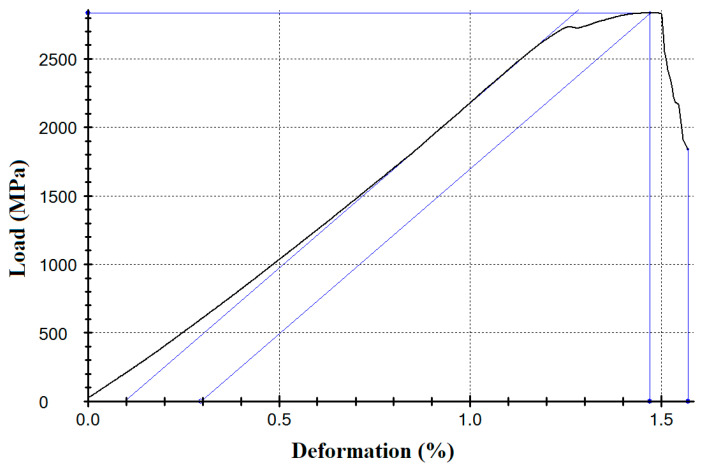
Load–strain diagram of a carbon fiber impregnated with EVA 28-40 (220 °C).

In the load–strain diagrams shown in Figure 7, three regions emerge: initial elasticity, transitional elasticity, and ultimate elasticity. In the initial region, the combined deformation of the carbon fibers and polymer occurs. Upon reaching a stress of approximately 0.3%, plastic flow of the polymer matrix begins in the spaces between the primary carbon fiber bundles of the filaments. This process equalizes the tension of the individual bundles and leads to an increase in the effective modulus of elasticity, which remains constant in the second linear region until failure and approaches the nominal value of the carbon fibers used.

Close relationships were observed previously for unidirectional carbon fibers impregnated with polysulfone by the solution method. Two nonlinear regions were observed in the load–strain diagrams [59]. The nonlinearity of the initial section is associated with the sliding of the bundles of filaments of the fiber relative to each other, leading to the orientation of the filaments in the direction of the load application, accompanied by an increase in the elastic modulus. As it was revealed in [59] using the SEM method, at low loading rates, elementary filaments inside the impregnated fiber are able to align themselves along the load application axis because a thermoplastic matrix can flow under the tensile stress force. The second nonlinear section occurs at high stresses and is caused by the destruction of the extremely elongated polymer interlayers, which leads to a further increase in the elastic modulus [59]. In a later paper [60], using the digital image correlation method, we confirmed that the deformation behavior of carbon fiber–polysulfone composites is provided by the ability of the thermoplastic matrix to deform under the load, which leads to a partial reorientation of individual bundles of carbon fibers. The onset of yielding of the polymer material leads to the appearance of a nonlinear section in the strain–stress curve [60].

**Figure 8:**
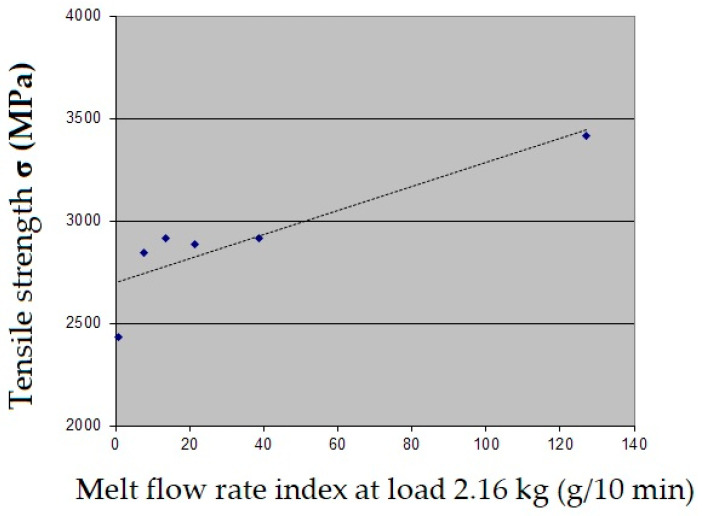
Correlation between tensile strength and MFI values for samples under investigation.

The increase in the melt flow index indicates that the degree of fiber strength realization is enhanced due to well filament penetration with polymer melts in the coextrusion system. The relationship between tensile strength and MVI magnitudes, given in Figure 8, shows the correlation between these two parameters. The highest tensile strength values were obtained for carbon filaments impregnated with EVA 28-40 extruded at 220 °C, with a MFI of 127 g/10 min. For polypropylene PP H030, the tensile strength is significantly lower at 2340 MPa, with an MFI value of 2.8 g/10 min. Furthermore, the elastic modulus values, both in the initial stage of loading and the effective modulus in the final stage of loading, do not depend on the polymer type and the flow index. This suggests that the long fiber path length in the polymer melt, with two passes through calibrating nozzles, ensures a roughly equal level of orientation of the elementary carbon fibers in the filaments.

## 4. Conclusions

We present a coextrusion method for producing 3D-printed filaments composed of continuous carbon fibers and thermoplastic polymers. This method enables the immediate production of filaments using a wide range of thermoplastic polymers and 12 K carbon fibers prior to printing. The filament path length in the polymer melt, combined with two calibrated dies, produces filaments with well-oriented basic carbon fibers, exhibiting an elastic modulus of 220–235 GPa. It has been shown that the initial carbon fiber strength in the filaments used for printing depends on the melt flow rate of the polymer used at the extrusion temperature, increasing with increasing MFI. For PP H030 polypropylene at a flow rate of 2.8 g/10 min, the filament tensile strength is 2432 MPa, while for EVA28-40 at 220 °C, with an MFI of 127 g/10 min, it increases to 3414 MPa. The selected extrusion temperatures of 190 and 220 °C ensure mild oxidation of polypropylene, polyethylene vinyl acetate, and their blends. No additional peaks were observed in the FTIR spectrum due to increased oxygen content or the presence of new oxygen-containing groups.

It should be noted that for composite printing, it is desirable to use a dual-filament system to ensure an optimal polymer content of 35–40% by weight in the composite material; the design of such a scheme is a task of forthcoming investigation. We can conclude that an elaborated approach to the printing process expands the range of polymers we can print with and enables the use of pre-fabricated filaments, polymer granulates, and the most common and widely available 12 K carbon filaments, potentially even 24 K ones.

## Figures and Tables

**Table 1:** Melting and solidification temperatures and melt flow indexes of the used polymers.

Polymer		PP H030	PP H270	EVA 28-40
Melting peak point. °C		166.7 ± 0.2	162.7 ± 0.3	67.1 ± 0.2
Crystallization peak point. °C		113.2 ± 0.3	120.2 ± 0.3	48.2 ± 0.3
Melt flow rate index. MVR g/10 min (load 2.16 kg) at temperature:	190 °C	0.9 ± 0.2	7.7 ± 0.3	38.9 ± 0.6
	230 °C	2.8 ± 0.2	27.6 ± 0.2	127 ± 3.3

**Table 2:** Tensile properties (elastic modulus E, tensile strength σ, elongation at break ε) and fiber content X for composite carbon filaments impregnated with thermoplastics.

Matrix Polymer	E, GPa	σ, MPa	ε, %	X, % wt. %	P, %
PP H030 GP (extruded at 190 °C)	228 ± 2.7	2432 ± 165	1.16 ± 0.05	73.2 ± 1.7	11.3 ± 0.4
PP H270 FF (extruded at 190 °C)	225 ± 1.7	2846 ± 243	1.49 ± 0.12	76.2 ± 0.9	13.5 ± 0.5
EVA 28-40 (extruded at 220 °C)	230 ± 2.5	3414 ± 176	1.58 ± 0.10	70.5 ± 1.3	12.3 ± 0.4
EVA 28-40 (extruded at 190 °C)	221 ± 1.3	2916 ± 137	1.38 ± 0.07	80.1 ± 0.9	11.7 ± 0.6
PP H270 FF/EVA 28-40 blend 50/50 (extruded at 190 °C)	220 ± 2.1	2884 ± 159	1.41 ± 0.11	79.1 ± 1.0	13.4 ± 0.3
PP H270 FF/EVA 28-40 blend 75/25 (extruded at 190 °C)	224 ± 2.4	2916 ± 151	1.39 ± 0.10	81.2 ± 1.1	11.8 ± 0.5

## Data Availability

The original contributions presented in this study are included in the article. Further inquiries can be directed to the corresponding author.
